# Supplementary figures and images for: ALDH1A3 Regulates Cellular Senescence and Senescence-Associated Secretome in Prostate Cancer
Source: Cancers (Basel). 2025 Mar 31;17(7):1184. doi: 10.3390/cancers17071184 (PMC11987895; doi:10.3390/cancers17071184)

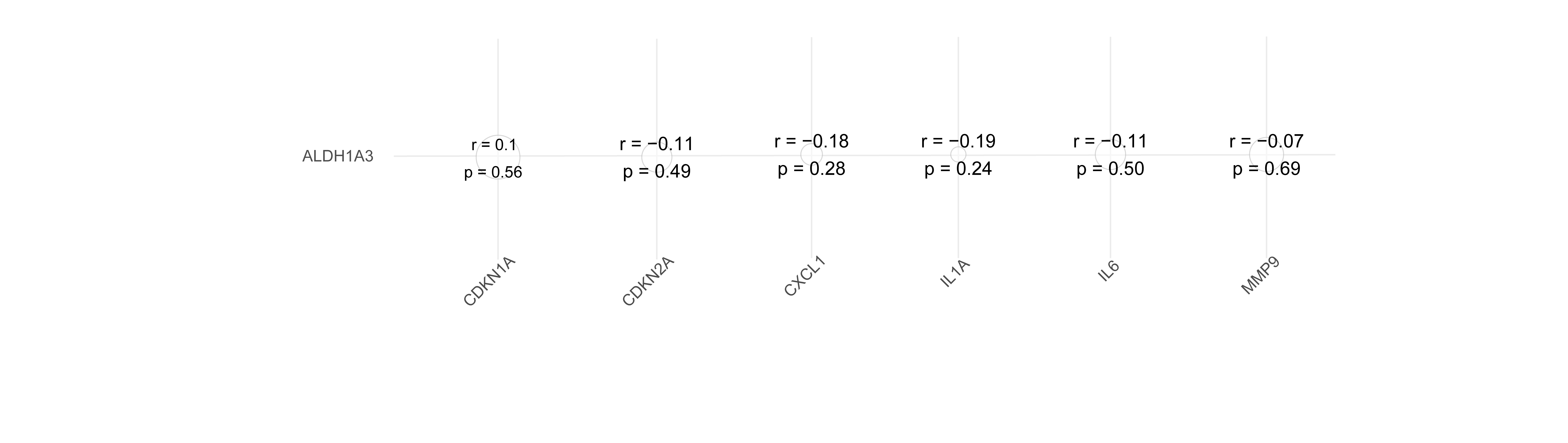

Supplement: Supplementary file 1 [file cancers-17-01184-s001.zip › Figure S1.tif]
